# Magnetoencephalography recordings reveal the spatiotemporal dynamics of recognition memory for complex versus simple auditory sequences

**DOI:** 10.1038/s42003-022-04217-8

**Published:** 2022-11-19

**Authors:** Gemma Fernández-Rubio, Elvira Brattico, Sonja A. Kotz, Morten L. Kringelbach, Peter Vuust, Leonardo Bonetti

**Affiliations:** 1 Center for Music in the Brain, Department of Clinical Medicine, Aarhus University & The Royal Academy of Music, Aarhus/Aalborg, Aarhus, Denmark; 2 Department of Neuropsychology and Psychopharmacology, Faculty of Psychology and Neuroscience, Maastricht University, Maastricht, The Netherlands; 3 Department of Education, Psychology, Communication, University of Bari Aldo Moro, Bari, Italy; 4 Centre for Eudaimonia and Human Flourishing, Linacre College, University of Oxford, Oxford, United Kingdom; 5 Department of Psychiatry, University of Oxford, Oxford, United Kingdom

**Keywords:** Cognitive neuroscience, Auditory system, Learning and memory

## Abstract

Auditory recognition is a crucial cognitive process that relies on the organization of single elements over time. However, little is known about the spatiotemporal dynamics underlying the conscious recognition of auditory sequences varying in complexity. To study this, we asked 71 participants to learn and recognize simple tonal musical sequences and matched complex atonal sequences while their brain activity was recorded using magnetoencephalography (MEG). Results reveal qualitative changes in neural activity dependent on stimulus complexity: recognition of tonal sequences engages hippocampal and cingulate areas, whereas recognition of atonal sequences mainly activates the auditory processing network. Our findings reveal the involvement of a cortico-subcortical brain network for auditory recognition and support the idea that stimulus complexity qualitatively alters the neural pathways of recognition memory.

## Abstract (web summary)

A magnetoencephalography study reveals that tonal musical sequences recruit neural activity in the hippocampus and anterior cingulate cortex, while atonal sequences activate auditory regions, suggesting the involvement of a cortico-subcortical brain network in auditory recognition.

## Introduction

Encoding and recognizing sounds that are structurally complex is a cognitive challenge relying on neural mechanisms that are not yet fully elucidated. To memorize complex sound sequences, we likely depend on the temporal organization of a stimulus’ components and memory functions^1^.

Memory encoding takes place in the hippocampus^2–4^, whereas subsequent processes related to recognition memory are supported by a functional network of interconnected regions in the medial temporal lobe, including the hippocampus, insula, and inferior temporal cortex^2,5,6^. For memory consolidation, communication between hippocampal and neocortical areas is needed^7–9^. Much evidence comes from studies using static visual stimuli, such as pictures of objects, faces, or natural scenes^10–12^. However, information and meaning also unfold over time as the brain attempts to predict upcoming stimuli based on prior memory representations. Hence, to better understand memory recognition and its underlying fast brain dynamics, novel methods must be adopted that highlight the temporal properties of dynamic stimuli. This can be done by studying the neural activity underlying the processing of sound sequences that acquire meaning through their evolution over time, such as music^13–15^.

Few neuroscientific studies have explored the neural underpinnings of musical memory, as reviewed by Campo and Brattico^16^. For example, using functional resonance imaging (fMRI) and a naturalistic music listening paradigm, Alluri et al.^17^ investigated the neural correlates of music processing and reported activation of cognitive, motor, and limbic brain networks for the continuous processing of timbral, tonal, and rhythmic features. Subsequently, using the same stimuli, Burunat et al.^18^ reported the recruitment of memory-related and motor brain regions during the recognition of musical motifs. Despite their contributions, these studies fail to identify the fine-grained temporal mechanisms of sound encoding and memory processes.

More recently, we combined the high temporal resolution of magnetoencephalography (MEG) with the high spatial resolution of magnetic resonance imaging (MRI) to study music recognition. These studies accentuated the temporal involvement of a widespread cortico-subcortical brain network comprising the primary auditory cortex, superior temporal gyrus, frontal operculum, cingulate gyrus, orbitofrontal cortex, and hippocampus during recognition of auditory (musical) sequences^19–21^. Overall, these investigations have provided unique insight into the neural mechanisms underlying the recognition of temporal sequences. What remains to be addressed is how these mechanisms are modulated by stimulus complexity.

Here, we used melodic sequences, where meaning emerged from the sequential combination of individual tones over time^21^, and varied the tone distribution to obtain new, complex musical sequences. In this scenario, encoding and recognition of the musical sequences largely depend on the sequential order of the tones that comprise it. We first selected musical sequences based on the rules of tonality, which is the dominant musical system in Western popular music^22^. Second, by modifying the tone intervals (i.e., the distances between pitches) while keeping all other variables (e.g., rhythm, tempo, timbre) constant, we generated matched atonal musical sequences. The stimulus manipulation was based on previous literature, which reported that tonal rather than atonal musical sequences are overall easier to process^23–27^ and more appreciated by non-expert listeners^27–29^. Unlike tonal music, atonal music is characterized by the absence of a clear tonal center and hierarchical stability, which significantly reduces its predictive value and gives rise to increased prediction errors^23–26,30^. Thus, we expected that the alteration of tonal intervals would reduce the predictability of the atonal sequences, leading to increased difficulty to recognize them.

To summarize, in the current study we used MEG and a musical recognition task^19–21^ while participants listened to and recognized auditory (musical) sequences of varying complexity and aimed at describing the fine-grained spatiotemporal dynamics of auditory recognition memory. Following the previous studies^17–21^, we expected that the recognition of auditory sequences would activate a widespread brain network that includes both auditory (e.g., primary auditory cortex, superior temporal gyrus, Heschl’s gyrus, planum temporale, insula) and memory processing areas (e.g., hippocampus, cingulate gyrus). We further hypothesized that neural activity would be distributed along two main frequency bands that reflected the occurrence of two different cognitive processes: a slow frequency band related to the recognition of the full musical sequence in memory processing areas, and a faster frequency band associated with the processing of each individual tone of the musical sequence in auditory regions. More importantly, we hypothesized that, based on stimulus complexity, tonal music would be more efficiently processed than atonal music, which would be reflected in different behavioral responses and distinct neural pathways during recognition of tonal and atonal sequences.

Our behavioral and neural results showed clear differences between the recognition of tonal and atonal sequences. Source reconstruction analyses indicated different activation clusters for tonal and atonal sequences. Overall, the neural activity was stronger in memory processing areas for memorized tonal sequences and in auditory processing regions for memorized atonal sequences.

## Results

### Behavioral data

**Fig. 1:**
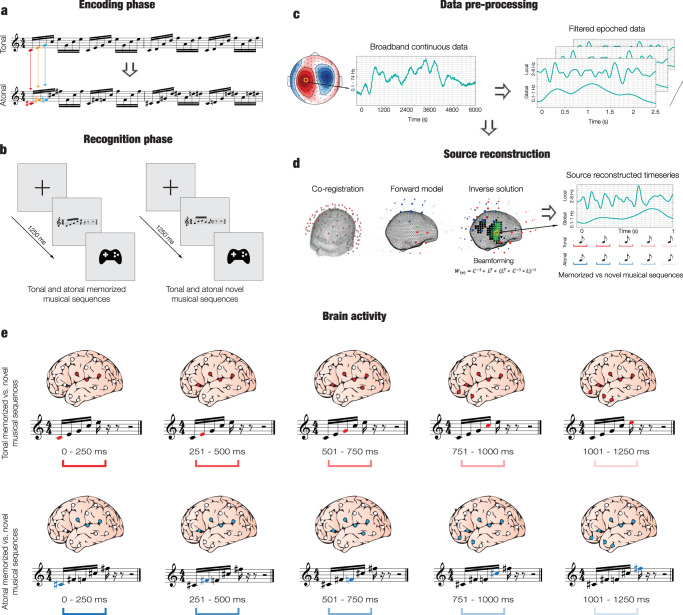
Experimental stimuli and design, data analyses, and (temporal) brain activity. **a** Two musical pieces were used in the experiment: the right-hand part of J. S. Bach’s Prelude No. 1 in C Major, BWV 846 (i.e., tonal, top row), and an atonal version of the prelude (i.e., atonal, bottom row). Both pieces were matched in terms of the sequential presentation of the tones, rhythmic patterns, dynamics, and duration, and their melodic contour was almost identical. The atonal piece was created by LB by assigning new tones that were one or two semitones lower or higher than the original tones of the tonal piece. For example, C (in red) was converted into C sharp (in red), E (in orange) was converted into F sharp (in orange), G (in blue) was converted into F (in blue), etc. **b** Participants (*n* = 71) performed the experimental task twice (once for the tonal piece and once for the atonal piece) and the order of presentation was randomized across participants. After listening to the full piece, participants were presented with excerpts that belonged to the piece or with new excerpts and were asked to state whether the excerpts were memorized or novel using a joystick. **c** The task was administered to the participants while their brain activity was recorded using MEG. The continuous neural data was preprocessed. **d** Source reconstruction analyses were conducted to identify the brain sources that generated the neural activity. The data was first bandpass-filtered into two frequency bands (0.1–1 Hz and 2–8 Hz) and the MEG and MRI data were co-registered. An overlapping-spheres forward model was computed using an 8-mm grid and a beamforming algorithm was applied as the inverse solution. Finally, the source reconstructed time series was computed for both tonal and atonal data and their contrast in both frequency bands. **e** Contrasts between memorized and novel sequences were calculated for each tone that comprised the tonal and atonal musical sequences for both frequency bands.

Participants performed an old/new auditory recognition task (Fig. 1). They first listened to a full musical piece (encoding) and subsequently identified which musical sequences were memorized or novel. During recognition, the response accuracy and reaction time of the participants were recorded using a joystick. These behavioral data were statistically analyzed to examine the differences between the four experimental conditions (memorized tonal sequences, novel tonal sequences, memorized atonal sequences, novel atonal sequences).

**Fig. 2:**
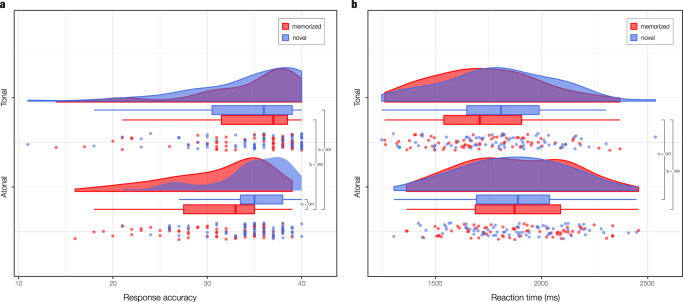
Analyses of behavioral data. Raincloud plots show the overlapping distributions of memorized and novel sequences in response accuracy (**a**) and reaction time (**b**; in milliseconds). Boxplots show the median and interquartile (IQR, 25–75%) range, whiskers depict the 1.5*IQR from the quartile, dots represent the individual data points, and asterisks denote a statistically significant difference between two conditions (*α* = 0.05). The analysis involved *n* = 71 participants.

A one-way analysis of variance (ANOVA) showed that the differences in response accuracy were statistically significant, *F*(3, 280) = 6.87, *p* = 0.002. Post-hoc analyses indicated that the average number of correct responses was significantly lower for memorized atonal sequences (M = 30.98, SD = 5.46) than for novel atonal (M = 34.51, SD = 4.26, *p* < 0.001), memorized tonal (M = 34.34, SD = 5.95, *p* = 0.002) and novel tonal sequences (M = 34.41, SD = 6.04, *p* = 0.001). Figure 2a shows the differences between conditions using raincloud plots^31^.

Regarding the mean reaction time, there was a statistically significant difference between conditions as determined by one-way ANOVA, *F*(3, 280) = 4.94, *p* = 0.002. Post-hoc analyses revealed that the average reaction time was significantly lower for memorized tonal sequences (M = 1735.17, SD = 259.91) compared to memorized atonal (M = 1879.44, SD = 259.34, *p* = 0.005) and novel atonal sequences (M = 1873.78, SD = 250.48, *p* = 0.007), but not compared to novel tonal sequences (M = 1799.52, SD = 267.14, *p* = .450). Figure 2b displays the differences between conditions.

Numerical source data for behavioral responses is provided in Supplementary Data 1.

### MEG sensor data

The MEG data (204 planar gradiometers and 102 magnetometers) were analyzed at the MEG sensor level, using the broadband signal. Although the emphasis of the study lays in identifying the brain areas involved in recognizing tonal versus atonal musical sequences, the MEG sensor data were examined to assess whether the neural signal was significantly different for memorized than for novel trials and thus would corroborate the results of previous studies^19,20^.

After averaging the epoched data of correct trials for each experimental condition and combining the planar gradiometers, paired-samples *t* tests were performed to identify which condition (memorized or novel) generated a stronger neural signal for each time sample and MEG sensor. Cluster-based MCS were then calculated to correct for multiple comparisons. This was performed independently for both tonal and atonal data (see Methods for details).

First, paired-samples *t* tests (*α* = 0.01) were calculated for the tonal data in the time interval 0–2500 ms (from the onset of the trial) using combined planar gradiometers as these sensors are less affected by external noise than magnetometers^32^. Next, multiple comparisons were corrected by using cluster-based MCS on the significant *t* tests’ results (*α* = 0.001, 1000 permutations). Three main significant clusters of activity were identified in three specific time intervals when contrasting memorized versus novel sequences, as reported in Table 1, Supplementary Fig. 1, and Supplementary Data 2. Additionally, two main significant clusters of activity were detected when contrasting novel versus memorized sequences (Table 1, Supplementary Fig. 2, and Supplementary Data 2).

**Table 1:** Significant clusters of activity for the tonal MEG sensor data.

Cluster number	Size	MEG channels	Time interval (seconds)	*p* value
Memorized versus novel tonal sequences				
1	224	63	0.14–0.187	<0.001
2	180	29	0.987–1.153	<0.001
3	150	37	0.807–0.887	<0.001
Novel versus memorized tonal sequences				
1	277	36	0.64–0.8	<0.001
2	242	30	0.38–0.513	<0.001

Regarding the atonal data, paired-samples *t* tests (*α* = 0.01) were calculated in the same time interval (0–2500 ms) using combined planar gradiometers. Next, multiple comparisons were corrected for using MCS on the significant *t* tests’ results (*α* = 0.001, 1000 permutations). This procedure identified three main significant clusters of activation when contrasting memorized versus novel excerpts (Table 2, Supplementary Fig. 3, and Supplementary Data 2). In the case of the novel versus memorized contrast, three main significant clusters of activity were found (Table 2, Supplementary Fig. 4, and Supplementary Data 2).

**Table 2:** Significant clusters of activity for the tonal MEG sensor data.

Cluster number	Size	Channels	Time interval (seconds)	*p* value
Memorized versus novel atonal sequences				
1	288	40	0.68–0.9	<0.001
2	215	44	0.52–0.66	<0.001
3	135	40	0.133–0.187	<0.001
Novel versus memorized atonal sequences				
1	478	52	1.167–1.267	<0.001
2	345	42	0.893–0.987	<0.001
3	320	44	0.653–0.74	<0.001

### Source reconstruction

After examining the strength of the neural signals at the MEG sensor level, we focused on the main aim of the study, namely to investigate the neural differences underlying the recognition of tonal versus atonal musical sequences in MEG reconstructed source space. To perform this analysis, we localized the brain sources of the event-related fields recorded by the MEG channels. This was performed for both the tonal and atonal sequences and for two frequency bands (slow [0.1–1 Hz] and faster [2–8 Hz]) that were used in Bonetti et al.^19,20^ and linked to the processing of single components (slow) relative to a whole sequence (fast). Additional analyses were computed to contrast the brain activity between the slow and fast frequency bands and to examine the pattern of activity at the standard delta (1–4 Hz) and theta (5–8 Hz) frequency bands.

### Slow frequency band (0.1–1 Hz)

The neural sources were calculated using a beamformer approach. First, a forward model was computed by considering each brain source as an active dipole and calculating its strength across the MEG sensors. Second, a beamforming algorithm was used as an inverse model to estimate the brain sources of neural activity based on the MEG recordings.

After computing the neural sources, a GLM was calculated at each timepoint and dipole location. A series of *t* tests (*α* = 0.05) was carried out at the first and group levels to estimate the main effect of memorized and novel conditions and their contrast for both the tonal and atonal data independently. Cluster-based MCS (*α* = 0.001, 1000 permutations) were computed to correct for multiple comparisons and to determine the brain activity underlying the development of the musical sequences. These analyses were carried out for five specific time intervals that corresponded to each of the tones comprising the sequences: first tone (0–250 ms), second tone (251–500 ms), third tone (501–750 ms), fourth tone (751–1000 ms), and fifth tone (1001–1250 ms). This was estimated for the memorized versus novel contrast for both tonal and atonal sequences independently and for memorized tonal versus memorized atonal sequences.

**Fig. 3:**
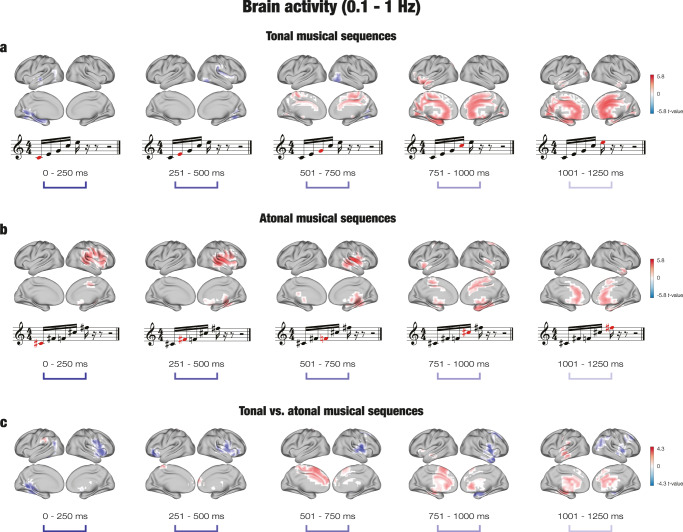
Brain activity underlying the recognition of musical sequences at the slow frequency band (0.1–1 Hz). **a** For tonal sequences, the brain activity was stronger for memorized (in red) than novel (in blue) sequences, particularly for the third (501–750 ms), fourth (751–1000 ms), and fifth (1001–1250) tones. The difference was localized in memory processing areas such as the cingulate gyrus, hippocampus, and parahippocampal gyrus. The analysis involved *n* = 71 participants. **b** For atonal sequences, the brain activity was stronger for memorized than novel sequences for all tones. The difference was mainly localized in auditory processing areas (e.g., superior temporal gyrus, Heschl’s gyrus) for the first three tones, and in memory processing areas (e.g., parahippocampal gyrus, hippocampus) for the fourth and fifth tones. The analysis involved *n* = 71 participants. **c** For the contrast between memorized tonal and atonal sequences, the brain activity was localized in memory processing areas for tonal sequences (in red), particularly for the last three tones, and in auditory processing areas for atonal sequences (in blue) for all tones. The analysis involved *n* = 71 participants.

Significant clusters of activity (*p* < 0.001) were located across a number of brain voxels (*k*) for each tone of the tonal sequences, as reported in Supplementary Data 3. For memorized tonal sequences, the neural activity was overall stronger for the third (*k* = 69), fourth (*k* = 266), and fifth tones (*k* = 229). The largest differences were localized in the middle cingulate gyrus, right supplementary motor area, precuneus, and left lingual gyrus for the third tone; the left amygdala, left parahippocampal gyrus, left lingual gyrus, left hippocampus, and middle cingulate gyrus for the fourth tone, and the anterior and middle cingulate gyrus and left lingual gyrus for the last tone. For novel tonal sequences, the brain activity was stronger for the first (*k* = 54) and second tones (*k* = 29). In particular, the difference between novel and memorized sequences was strongest in the left calcarine fissure, left lingual gyrus, left hippocampus, left precuneus, and left superior temporal gyrus for the first tone, and the right fusiform gyrus, right lingual gyrus, and right inferior occipital gyrus for the second tone. The contrast between memorized and novel tonal sequences for the slow band is depicted in Fig. 3a.

In the case of atonal sequences, significant activity clusters were found primarily in auditory and medial temporal lobe regions in the right hemisphere for memorized sequences, and the neural activity was stronger for memorized than novel sequences across all five tones (*k*_*1*_ = 132, *k*_*2*_ = 163, *k*_*3*_ = 130, *k*_*4*_ = 140, *k*_*5*_ = 64), as reported in Supplementary Data 3. In particular, the brain activity was strongest in the right Rolandic operculum, right superior temporal gyrus, right Heschl’s gyrus, right supramarginal gyrus, and right insula for the first tone; the right Heschl’s gyrus, right superior temporal gyrus, right Rolandic operculum, right middle temporal gyrus, and right insula for the second tone; the right putamen, right insula, right Rolandic operculum, right Heschl’s gyrus, and right thalamus for the third tone; the parahippocampal gyrus, right fusiform gyrus, right hippocampus, and putamen for the fourth tone; and the anterior cingulate cortex, middle frontal gyrus, and caudate nucleus for the last tone. No significant clusters of activity were located in the slow band for novel atonal sequences. Figure 3b pictures the contrast between memorized and novel atonal sequences in the slow band.

Finally, to examine the differences during recognition of tonal versus atonal sequences, the brain activity during recognition of memorized tonal and memorized atonal sequences was contrasted. Significant activity clusters were located for both types of musical sequences across all tones (see Supplementary Data 3). For tonal sequences, the number of significant voxels was higher for the third (*k* = 70) and fifth tones (*k* = 79), whereas for atonal sequences the number of significant brain voxels was higher for the first (*k* = 98), second (*k* = 80), and fourth tones (*k* = 103). Overall, the brain activity was stronger in left cingulate and medial temporal lobe regions for tonal sequences, and in right auditory processing areas for atonal sequences. Specifically, in the case of memorized tonal sequences, the neural activity was localized in the supplementary motor area, left median cingulate gyrus, and superior frontal gyrus for the third tone, and the left hippocampus, left superior temporal gyrus, left thalamus, left insula, left putamen, and left parahippocampal gyrus for the fifth tone. For memorized atonal sequences, the neural activity was localized in the left lingual gyrus, left precuneus, left calcarine fissure, middle temporal gyrus, and right insula at the first tone; the inferior frontal gyrus, right precentral gyrus, right Rolandic operculum, and right superior temporal gyrus for the second tone; the right Rolandic operculum, right middle frontal gyrus, right postcentral gyrus, right putamen, and right insula for the fourth tone; and the right middle frontal gyrus, right angular gyrus, and right thalamus for the last tone. The contrast between memorized tonal and atonal sequences in the slow band is shown in Fig. 3c.

### Faster frequency band (2–8 Hz)

The same procedure was carried out for assessing the brain activity underlying the recognition of musical sequences in the faster frequency band (2–8 Hz). Once the GLM was computed, cluster-based MCS (*α* = 0.001, 1000 permutations) were calculated for five time intervals corresponding to each of the five tones that formed the sequence. Again, this was estimated for the memorized versus novel contrast for both tonal and atonal sequences and memorized tonal versus memorized atonal sequences.

**Fig. 4:**
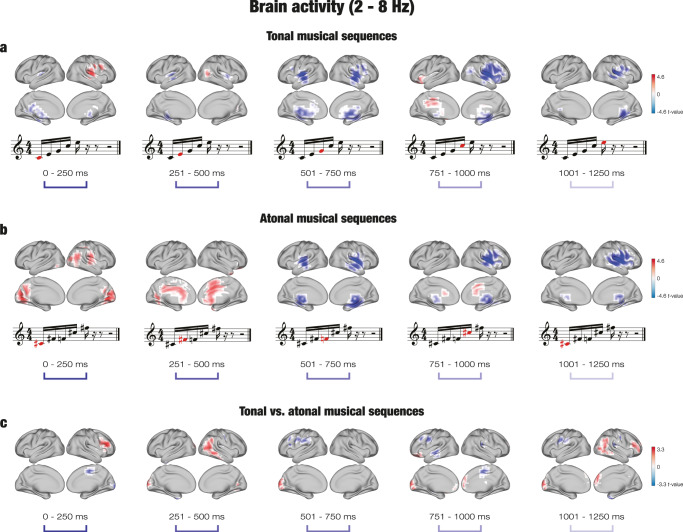
Brain activity underlying the recognition of musical sequences at the faster frequency band (2–8 Hz). **a** For tonal sequences, the brain activity was stronger for novel (in blue) than memorized (in red) sequences, particularly for the last three tones. The difference was localized in auditory processing areas such as Heschl’s gyrus, superior temporal gyrus, and Rolandic operculum. The brain activity was stronger for memorized than novel sequences for the first, second and fourth tones in areas such as the Rolandic operculum (tone 1), occipital gyrus (tone 2) and inferior frontal gyrus (tone 4). The analysis involved *n* = 71 participants. **b** For atonal sequences, the brain activity was stronger for novel than memorized sequences for the last three tones in areas such as the insula, Heschl’s gyrus, and superior temporal gyrus. The brain activity was stronger for memorized than novel sequences for the first two tones in the calcarine fissure and cingulate gyrus. The analysis involved *n* = 71 participants. **c** For the contrast between tonal and atonal sequences, the brain activity was mostly scattered and weak, but the neural activity was stronger in the frontal gyrus (tones 1, 4, and 5), temporal gyrus (tones 2 and 3), and occipital gyrus (tones 2–5) for tonal memorized sequences (in red), and in the supplementary motor area (tone 1), frontal gyrus (tones 3 and 4), middle temporal gyrus (tone 4) and postcentral gyrus (tone 5) for atonal memorized sequences (in blue). The analysis involved *n* = 71 participants.

Regarding the contrast for tonal sequences, significant clusters of activity (*p* < 0.001) were located in multiple brain voxels for both memorized and novel sequences, as reported in Supplementary Data 3. For memorized tonal sequences, the neural activity was overall stronger for the first tone (*k* = 74), whereas it was stronger for novel tonal sequences for the second (*k* = 36), third (*k* = 200), fourth (*k* = 196), and fifth tones (*k* = 70). The brain activity was localized in the right Rolandic operculum, right insula, right Heschl’s gyrus, and right superior temporal gyrus for the first tone for memorized tonal sequences. For novel tonal sequences, the main active areas were the left superior temporal gyrus, insula, Heschl’s gyrus, and left hippocampus for the second tone; Heschl’s gyrus, superior temporal gyrus, insula, and putamen (k = 11) for the third tone; right Heschl’s gyrus, right insula, right Rolandic operculum, and right superior temporal gyrus for the fourth tone; and the right Rolandic operculum, right Heschl’s gyrus, right hippocampus, and right thalamus for the fifth tone. Figure 4a displays the contrast between memorized and novel tonal sequences.

For the contrast between memorized and novel atonal sequences, the majority of significant clusters of activity were localized at the first (*k* = 166) and second tones (*k* = 104) for memorized sequences, and at the third (*k* = 189), fourth (*k* = 118), and fifth tones (*k* = 156) for novel sequences, as reported in Supplementary Data 3. For memorized atonal sequences, the neural activity was strongest at the calcarine fissure, lingual gyrus, and right Rolandic operculum for the first tone, and the cingulate gyrus, right supplementary motor area, and superior frontal gyrus for the second tone. For novel atonal sequences, the neural activity was strongest at the insula, putamen, superior temporal gyrus, and Heschl’s gyrus for the third tone; the right insula, right putamen, right Heschl’s gyrus, right Rolandic operculum, and right superior temporal gyrus for the fourth tone; and the right insula, right Rolandic operculum, right Heschl’s gyrus, right putamen, and right superior temporal gyrus for the fifth tone. The contrast between memorized and novel atonal sequences for the faster band is shown in Fig. 4b.

Finally, the significant clusters of activity in the tonal versus atonal contrast for the faster band are reported in Supplementary Data 3. In the case of memorized tonal sequences, the number of significant brian voxels was higher for the first (*k* = 20), second (*k* = 44), fourth (*k* = 45), and fifth tones (*k* = 71). The neural activity was located in the right inferior frontal gyrus and right middle frontal gyrus at the first tone; the right middle temporal gyrus, right inferior parietal gyrus, right angular gyrus, middle occipital gyrus, and left superior occipital gyrus at the second tone; the frontal gyrus for the fourth tone; and the right middle occipital gyrus, right frontal gyrus, and right middle temporal gyrus for the fifth tone. For memorized atonal sequences, the number of significant voxels was higher at the third tone (*k* = 38) and the neural activity was mainly localized in the left inferior frontal gyrus, left middle frontal gyrus, left supramarginal gyrus, and right supplementary motor area. Figure 4c shows the contrast between tonal and atonal sequences for the fast band.

Additional analyses were computed to calculate the difference in neural activity between the slow and faster frequency bands. The results are reported in Supplementary Fig. 5 and Supplementary Data 4.

Altogether, we found significant differences between tonal and atonal sequences, especially for the slow frequency band. Recognition of memorized tonal sequences elicited stronger neural activity in left medial temporal lobe and cingulate regions in the last three tones of the sequences, whereas recognition of memorized atonal sequences was supported by activation in right auditory regions from the second tone onwards.

### Delta (1–4 Hz) and theta (5–8 Hz) frequency bands

Finally, to obtain a complete picture of the brain activity underlying memory recognition of auditory sequences and to make our results comparable with previous literature, additional analyses were performed in two standard frequency bands: delta (1–4 Hz) and theta (5–8 Hz). The same procedure was carried out for assessing the brain activity underlying the recognition of musical sequences in each frequency band independently. Once the GLM was computed, cluster-based MCS (*α* = 0.001, 1000 permutations) were calculated for five time intervals, corresponding to each of the five tones that formed a sequence. Again, this was estimated for the memorized versus novel contrast for both tonal and atonal sequences. The significant clusters of activity for the 1–4 Hz and 5–8 Hz frequency bands are reported in Supplementary Data 5.

For the delta band, significant clusters of activity (*p* < 0.001) were located across a number of brain voxels (*k*) for each tone of the tonal sequences. For memorized tonal sequences, the neural activity was overall stronger at the first (*k* = 39), third (*k* = 72), and fourth tones (*k* = 92). The largest differences were localized in the left putamen, left lingual gyrus, left inferior frontal operculum, and right Heschl’s gyrus. For novel tonal sequences, the brain activity was stronger at the second tone (*k* = 129). In particular, the difference between novel and memorized sequences was strongest in the left superior temporal gyrus, right insula, and right putamen. For memorized atonal sequences, the neural activity was stronger at the first (*k* = 149), second (*k* = 167), and third (*k* = 50) tones. This activity was strongest in the right precuneus and right calcarine fissure. For novel atonal sequences, the fourth (*k* = 55) and fifth (*k* = 41) tones elicited more activity, particularly in the right Heschl’s gyrus and right Rolandic operculum. The contrast for the 1–4 Hz band is shown in Supplementary Fig. 6.

In the case of the theta band, we found significant activity at the first (*k* = 47) tone of memorized tonal sequences, namely in the left inferior frontal gyrus and right Heschl’s gyrus. The neural activity was stronger for novel tonal sequences at the second (*k* = 13), third (*k* = 89), fourth (*k* = 19), and fifth (*k* = 161) tones. The largest differences were localized in the right inferior frontal gyrus, left caudate, and right insula. For memorized atonal sequences, the neural activity was stronger at the first (*k* = 129), second (*k* = 167), third (*k* = 50) and fourth (*k* = 93) tones. This activity was strongest at the left calcarine fissure and right precuneus. For novel atonal sequences, the neural activity was stronger at the fifth tone (*k* = 33), particularly in the right insula and right putamen. The contrast for the 1–4 Hz band is shown in Supplementary Fig. 7.

### Correlation with familiarity ratings

Data from the familiarity ratings regarding the tonal piece were correlated to the source analysis on the recognition of memorized tonal sequences to examine whether familiarity with the piece could predict neural activity during recognition.

Minor (*t* value <0.4) but significant positive correlations were found between familiarity ratings and activation in the left middle temporal gyrus and left middle occipital gyrus for the last three tones of the memorized tonal sequences. Detailed statistics and a graphical depiction of the results are reported in supplementary materials (Supplementary Data 6 and Supplementary Fig. 8).

## Discussion

This study set out to investigate the brain activation underlying the recognition of auditory musical sequences characterized by different levels of complexity (tonal and atonal). Behavioral data showed clear differences between the recognition of tonal and atonal sequences and significant clusters of activation were observed at the MEG sensor level. Source reconstruction analyses indicated different activation clusters for tonal and atonal sequences, particularly in the slow frequency band. Overall, the neural activity was stronger in memory processing areas for memorized tonal sequences and in auditory processing regions for memorized atonal sequences.

Prior to focusing on the differences in brain activity related to distinct levels of recognition complexity, we verified that the current results were consistent with previous studies. Indeed, the brain areas activated during the recognition of tonal sequences confirmed the involvement of a widespread brain network including both auditory and memory processing regions^17–19,21,33,34^. Specifically, we observed increased activation in auditory regions, such as the superior temporal gyrus^35^ and Heschl’s gyrus^36^, and in the cingulate gyrus^37^ and medial temporal lobe structures (hippocampus, parahippocampal cortex) associated with memory recognition^10,38,39^. Furthermore, in accordance with previous research, the neural activity was clearly distributed in two frequency bands^19,20^. The slow band (0.1–1 Hz) was linked to the recognition of the whole auditory sequences (global processing), which was reflected by the stronger activation occurring in this band for the recognition of the memorized sequences. Conversely, the faster band (2–8 Hz) was associated with the processing of the individual tones (local processing), as suggested by the stronger neural activity in auditory regions during processing of novel sequences.

Regarding the recognition of tonal and atonal sequences, we observed distinct neural pathways when processing and recognizing the two types of auditory stimuli. While the recognition of tonal sequences mainly recruited a widespread brain network involving cingulate gyrus and hippocampus in the right hemisphere, the recognition of atonal sequences was mainly associated with a sustained, slow activity in the left auditory cortex. These results can be interpreted in light of different theoretical frameworks, namely predictive coding of music^40,41^, harmonicity^42^, global neuronal workspace^43,44^, and temporo-spatial theory of consciousness^45,46^.

According to the predictive coding of music theory, the brain’s predictive model is being continuously updated while listening to music to decrease precision-weighted prediction errors^40,47,48^. Specifically, bottom-up sensations evoked by auditory stimuli are processed in primary cortices and contrasted with top–down predictions in higher-order cortices to generate musical expectations and minimize hierarchical prediction errors^48–50^. Predictive mechanisms rely on long- and short-term memory functions, familiarity, and listening strategies to create musical expectations^48^. This theory provides a framework for studying music perception^51–54^, training^55,56^, action^57,58^, synchronization^59–61^, and emotion^62–64^.

Previous research has shown the predictive value of atonal music is weaker than tonal music, increasing its complexity and prediction errors^23–27,30^. Additionally, this change in stimulus predictability undermines the processing^23–26^ and enjoyment^27–29^ of atonal music. This was apparent when examining the behavioral results of the current study, as memorized atonal sequences were more slowly and less accurately recognized. Furthermore, the distribution of the neural activity in two frequency bands suggests a combination of top–down predictions in the slow band, which is related to the recognition of memorized sequences, and bottom-up predictions in the faster band, as the prediction error increases with novel sequences. It is important to note that, while results can be interpreted in the framework of predictive coding, the current study did not aim to test the predictive coding of music theory (e.g., by measuring the prediction error generated during recognition of the musical sequences). Therefore, future studies could replicate and expand these results within the predictive coding of music framework.

An alternative explanation for these results focuses on the harmonicity of auditory stimuli. Tonal music has been closely linked to the harmonic series, a natural sequence of sound frequencies that are integer multiples of a fundamental. Environmental sounds are typically nonharmonic, whereas both human and animal vocalizations contain harmonic structures^42^. The tonotopic organization of the human auditory cortex is particularly sensitive to harmonic tones, suggesting that this region developed to process harmonics due to their relevance for social communication^65,66^. These results indicate that distinct neural pathways are activated when recognizing auditory stimuli that are not coherent with the natural harmonic series and thus arguably more complex to process. Indeed, we found that for memorized tonal sequences, the brain activity was primarily located at the cortico-hippocampal network in the right hemisphere, and for memorized atonal sequences the auditory network in the left hemisphere. As mentioned above, atonal music is more difficult to process^23–27^ and less appreciated^27–29^ than tonal music. Thus, to recognize atonal sequences, auditory regions might require additional processing of the sounds to extract the relevant information carried by the first tones of the sequences. Then, this information might be broadcasted to high-order areas for the recognition of the sequences. One possible approach to further test the harmonicity hypothesis in relation to sequence recognition would be to create a collection of pieces that are systematically varied in terms of their similarity to the natural harmonic series. Future studies are called to investigate such perspectives.

The current results are also consistent with the global neuronal workspace hypothesis^43,44,67,68^. According to this theory, stimuli become conscious when they ignite late, high-order regions in response to the activation of sensory cortices involved in perceptual representation. Conversely, unconscious information does not reach high-processing brain areas and neural activity is limited to sensory cortices^44,67,69^. Importantly, we found that tonal sequences induced a late and robust activation of memory processing regions. Although it is unclear why atonal sequences were differently processed by the brain, we can confirm that the complexity of the stimuli modulates the transition from primary sensory areas to the global neuronal workspace, providing additional information to this comprehensive theoretical framework.

Finally, our results are in line with the temporo-spatial theory of consciousness, which focuses on four spatiotemporal mechanisms of the brain activity (expansion, globalization, alignment, and nestedness) to account for four corresponding consciousness dimensions (phenomenal content, access, form/structure, and level/state)^45,46^. In the current and previous studies^19,20,70^, we found that activity in the slow frequency band (0.1–1 Hz) was linked to memory recognition of auditory (musical) temporal sequences. These results support the theory’s notion of a temporal receptive window that allows for the integration of stimulus sequences into one cognitive unity in slow frequency bands^45^. In both tonal and atonal memorized sequences, we found stronger activation in memory processing regions in the slow frequency band.

Although the aim of this study was memory recognition of auditory sequences extracted from musical pieces of varying complexity, it would be interesting to examine the differences in brain activity between encoding and recognition of simple and complex auditory sequences. In the current study, the recognition task included 160 five-tone auditory sequences, whereas the complete musical pieces were played only four times in the encoding task, which prevents direct comparison between the two. In an attempt to link the encoding and recognition phases, we computed correlation analyses between the familiarity ratings provided by participants and the brain activity underlying the recognition of tonal memorized sequences. Greater familiarity with the tonal piece was correlated with increased activation in the middle temporal gyrus and middle occipital gyrus in the left hemisphere for the last three tones of the memorized tonal sequences. This result supports previous studies linking these two regions to recognition memory^71^ and suggests that deep encoding of music plays a role in the recognition process. Future studies should replicate and expand the current results by examining the neural correlates underlying not only memory recognition but also memory encoding of auditory sequences of varying complexity.

The current study provides valuable insights into the brain mechanisms underlying the recognition of auditory sequences. The results are consistent with those of previous studies and evidence the engagement of a large brain network that comprises both memory processing and auditory regions when recognizing music. Results further highlight the importance of stimulus complexity for the processing of temporal sequences and hint that the brain employs different strategies to account for this complexity.

## Methods

### Participants

The participant sample consisted of 71 volunteers (38 males and 33 females) aged 18 to 42 years old (mean age: 25 ± 4.10 years). All participants were healthy and reported normal hearing. Participants were recruited in Denmark and came from Western countries with matching socioeconomic and educational backgrounds.

The project was approved by the Ethics Committee of the Central Denmark Region (De Videnskabsetiske Komitéer for Region Midtjylland, ref. 1-10-72-411-17). The experimental procedures were carried out in compliance with the Declaration of Helsinki—Ethical Principles for Medical Research. All participants gave informed consent before starting the experimental procedure.

### Experimental stimuli and design

Two musical compositions were used in the experiment: the right-hand part of Johann Sebastian Bach’s Prelude No. 1 in C Major, BWV 846 (hereafter referred to as the tonal piece), and an atonal version of the prelude (hereafter referred to as the atonal piece). MIDI versions were created using Finale (MakeMusic, Boulder, CO) and both pieces lasted 2.5 min each, with the same duration for all tones. LB composed the atonal piece based on the tonal piece. In particular, new tones were assigned to each of the tones comprising Bach’s original prelude. These new tones were one or two semitones higher or lower than the original tones, and the same tone conversion was applied throughout the entire tonal piece to obtain the atonal piece (e.g., every C tone in the tonal piece was converted into a C sharp in the atonal piece). Thus, both compositions were identical in terms of the sequential presentation of the tones (i.e., if C was positioned as 1st, 7th, and 8th tone in the tonal piece, C sharp occupied the same positions in the atonal piece), their rhythmic pattern, dynamics, and duration. Thus, the crucial difference between the two pieces was that the tonal piece was in the key of C Major, whereas the atonal piece did not have a musical key. The first two bars of each piece are displayed in Fig. 1a, showing similarities and correspondence between the two pieces.

Forty musical excerpts (i.e., short melodies or sequences) were extracted from each of the pieces. All excerpts consisted of the first five notes of each bar and lasted for 1250 ms (250 ms per note). In addition, 40 new excerpts were created for each piece based on the original ones. These new sequences were matched to the original ones among several variables, to prevent potential confounds. Specifically, they were matched for rhythm, volume, timbre, tempo, meter, and tonality.

To quantify the differences in complexity between tonal and atonal sequences, we computed information content (IC) and entropy (H) values for each tone of the musical sequences using the Information Dynamics of Music (IDyOM) model^56,72^. IC and H provide an estimation of the predictability of each tone and uncertainty with which it can be predicted^73^. The IC represents the minimum number of bits required to encode ${e}_{i}$ and is described by the Eq. (1):

1 $${IC}\left({e}_{i}|{e}_{\,\left(i-n\right)+1}^{i-1}\right)={{{{{{\rm{log }}}}}}}_{2}\frac{1}{p\left({e}_{i}|{e}_{\,\left(i-n\right)+1}^{i-1}\right)}$$

where $p({e}_{i}|{e}_{\,\left(i-n\right)+1}^{i-1})$ is the probability of the event ${e}_{i}$ given a previous set of ${e}_{\,\left(i-n\right)+1}^{i-1}$ events.

The H gives a measure of certainty/uncertainty about an upcoming event given the previous set of ${e}_{\,\left(i-n\right)+1}^{i-1}$ events and is calculated by the Eq. (2):

2 $$H\left({e}_{\,\left(i-n\right)+1}^{i-1}\right)=\mathop{\sum}\limits_{e\in A}p\left({e}_{i}|{e}_{\,\left(i-n\right)+1}^{i-1}\right){IC}\left({e}_{i}|{e}_{\,\left(i-n\right)+1}^{i-1}\right)$$

Equation (2) shows that, if the probability of a given event ${e}_{i}$ is 1, the probability of the other events in $A$ will be 0 and therefore $H$ will be equal to 0 (maximum certainty). Conversely, if all the events are equally likely, $H$ will be maximal (maximum uncertainty). Higher levels of IC and H indicate less predictability of an upcoming tone and therefore a higher level of complexity.

The ${{{{{\rm{IC}}}}}}$ and H of each note of the tonal and atonal sequences were calculated using a model trained on a corpus of prototypical Western musical pieces employed by IDyOM^56^. After obtaining the IC and H scores for the individual tones, we performed paired-samples *t* tests to compute the difference in IC and H between tonal and atonal sequences. There was a statistically significant difference in IC (*t*_73_ = −8.389, *p* < 0.001) and H (*t*_73_ = −3.374, *p* = 0.001) values between tonal and atonal sequences, meaning that atonal sequences (mean IC: 6.69 ± .86; mean H: 4.74 ± .17) are more complex than tonal ones (mean IC: 5.66 ± 0.83; mean H: 4.65 ± .11).

The stimuli were employed in an old/new auditory recognition paradigm that was administered to the participants while their brain activity was recorded using MEG (Fig. 1b). The paradigm consisted of two parts, encoding and recognition, and was performed twice, once for the tonal piece and once for the atonal piece. The order of tonal/atonal was counterbalanced across participants. During the encoding part, participants actively listened to four repetitions of the entire musical piece (tonal or atonal) and tried to memorize it as much as possible. Afterwards, they were presented with the 80 musical excerpts (40 memorized and 40 novel excerpts, randomly ordered) and stated whether the excerpts belonged to the piece they had previously listened to (memorized) or whether they were new excerpts (novel). Response accuracy and reaction time were recorded using a joystick.

### Behavioral data analysis

The behavioral data were statistically analyzed to examine the differences between the four experimental conditions (memorized tonal sequences, novel tonal sequences, memorized atonal sequences, novel atonal sequences) in response accuracy and reaction times. Two ANOVAs were computed (*α* = 0.05). Post-hoc analyses with Bonferroni correction (adjusted *α* = 0.0125) were used to compare each pair of experimental conditions.

### Neural data acquisition

The MEG recordings were acquired in a magnetically shielded room at Aarhus University Hospital (Denmark) with an Elekta Neuromag TRIUX MEG scanner with 306 channels (Elekta Neuromag, Helsinki, Finland). The data were recorded at a sampling rate of 1000 Hz with an analog filtering of 0.1–330 Hz. Before starting the recordings, the head shape of the participants and the position of four Head Position Indicator (HPI) coils with respect to three anatomical landmarks (nasion and fiducials) were registered using a 3D digitizer (Polhemus Fastrak, Colchester, VT, USA). This information was later used to co-register the MEG data with the MRI anatomical scans. During the MEG recordings, the HPI coils registered the continuous head localization, which was subsequently used for movement correction analyses. Additionally, two sets of bipolar electrodes were used to record eye movements and cardiac rhythm for later removing electrooculography (EOG) and electrocardiography (ECG) artifacts.

The MRI scans were recorded on a CE-approved 3 T Siemens MRI-scanner at Aarhus University Hospital (Denmark). The data were recorded using a structural T1 with a spatial resolution of 1.0 × 1.0 × 1.0 mm and the following sequence parameters: echo time (TE) = 2.96 ms, repetition time (TR) = 5000 ms, reconstructed matrix size = 256 × 256, bandwidth = 240 Hz/Px.

The MEG and MRI recordings were acquired in two separate sessions.

### Neural data pre-processing

The raw MEG sensor data (204 planar gradiometers and 102 magnetometers) were first preprocessed by MaxFilter^32^ in order to suppress external interferences. The following MaxFilter parameters were applied: spatiotemporal signal space separation (SSS), movement compensation using cHPI coils (default step size: 10 ms), down-sample from 1000 Hz to 250 Hz, correlation limit of 0.98 between inner and outer subspaces used to reject overlapping intersecting inner/outer signals during spatiotemporal SSS. In addition, the data were corrected for head motion and downsampled to 250 Hz. The data were then converted into Statistical Parametric Mapping (SPM)^74^ format and analyzed in MATLAB (MathWorks, Natick, MA, USA) with the Oxford Centre for Human Brain Activity (OHBA) Software Library (OSL) (https://ohba-analysis.github.io/osl-docs/), a freely available software that builds upon Fieldtrip^75^, FSL^76^, and SPM toolboxes. The signal was high-pass filtered (0.1 Hz of cutoff) to remove external frequencies and a notch filter was subsequently applied (48–52 Hz) to correct for inferences of the electric current. The signal was further downsampled to 150 Hz and the continuous MEG data were visually inspected to remove artifacts using the OSLview tool. An independent component analysis (ICA) was performed to remove EOG and ECG components. After reconstructing the signal with the remaining components^77^, the data were epoched into 160 trials (80 excerpts from each musical piece). Each trial lasted 1350 ms (1250 ms plus 100 ms of baseline time) and further analyses were performed on correctly identified trials only (see Fig. 1c).

### MEG sensor data analysis

The primary focus of this study was on detecting differences in the brain activity underlying the recognition of tonal versus atonal musical sequences. However, the data were first analyzed at the MEG sensor level to verify that the neural signal was stronger for memorized versus novel musical sequences. This first step was essential to replicate previous findings obtained using a very similar experimental setting and paradigm and thus assess the quality of our data^19,20^.

Following the pre-processing of the neural data, and in accordance with MEG analysis guidelines^78^, all trials belonging to one condition were averaged together. This procedure resulted in four mean trials: one for memorized trials and one for novel trials for each musical piece (i.e., memorized tonal, novel tonal, memorized atonal, novel atonal). Next, each pair of planar gradiometers was combined by a sum root square. Paired-samples *t* tests (*α* = 0.01) were then calculated to contrast the memorized and novel conditions for both the tonal and atonal pieces, independently. This was performed for each combined planar gradiometer and each timepoint in the time-range 0–2500 ms (from the onset of the first tone of the musical sequences) in order to determine which condition generated a stronger neural signal. The analyses were calculated for planar gradiometers, since these sensors are less affected by external noise and thus highly reliable when computing analyses at the MEG sensor level^78–81^. Multiple comparisons were corrected using cluster-based Monte Carlo simulations (MCS)^82^ (*α* = 0.001, 1000 permutations) on the significant *t* tests’ results. Specifically, for each timepoint, a 2D matrix was generated reproducing the spatial location of the MEG channels and the results of the *t* tests of each MEG channel binarized according to their *p* values (0 s for not significant tests and 1 s for significant tests [i.e., *p* < 0.01]). The elements of the resulting 3D matrix were then submitted to 1000 permutations. For each permutation, we identified the maximum cluster of permuted 1 s, and we built a reference distribution using the maximum cluster sizes detected for each of the 1000 permutations. Finally, the original clusters that had a larger size than 99.9% of the maximum cluster sizes of the permuted data were considered significant.

### Source reconstruction

After examining the strength of the neural signals at the MEG sensor level, we focused on the main aim of the study, which was to investigate the neural differences underlying the recognition of tonal versus atonal musical sequences in MEG reconstructed source space. To perform this analysis, we localized the brain sources of the neural signal recorded in the MEG channels. This procedure required designing a forward model, computing the inverse solution (in this case, using a beamforming approach), and identifying the statistically significant brain sources underlying the recognition of tonal and atonal sequences and their contrasts over time. Figure 1d shows the graphical depiction of the source reconstruction analyses.

Before computing the source reconstruction algorithm, the continuous data were bandpass filtered into two frequency bands: a slow band (0.1–1 Hz) and a faster band (2–8 Hz). The data collected and analyzed were event-related fields, not induced responses or oscillations. These bands were selected based on prior findings by Bonetti et al.^19,20^ which suggest that event-related fields in the faster band were responsible for a sensorial elaboration of each object (tone) in a sequence, while event-related fields in the slow band accounted for the recognition of the whole temporal sequence. To make it comparable with previous literature, additional analyses were computed at the delta (1–4 Hz) and theta (5–8 Hz) frequency bands. Finally, a contrast between the slow and faster frequency bands was calculated.

First, an overlapping-spheres forward model was computed using an 8-mm grid. This theoretical head model considers each brain source as an active dipole and describes how the unitary strength of such a dipole is reflected across the MEG sensors^83^. Using the information collected with the 3D digitizer, the MEG data and individual T1-weighted images were co-registered and the forward model was subsequently computed. An MNI152-T1 template with 8-mm spatial resolution was used in four cases in which the individual anatomical scans were not available. Second, a beamforming algorithm was employed as the inverse model. This is one of the most widely used algorithms for estimating the brain sources from MEG channels’ data and consists of utilizing a different set of weights which are sequentially applied to the source locations (dipoles) for isolating the contribution of each source to the activity recorded by the MEG channels for each timepoint^78,84,85^.

After estimating the brain sources of the signal recorded on the MEG channels, a General Linear Model (GLM) was estimated sequentially for each timepoint at each dipole location. At the first level, the main effect of memorized and novel conditions, as well as their contrast, was computed independently for each participant. At the group level, *t* tests were carried out for each dipole location to obtain the main effect of tonal, atonal, and their contrast computed on all aggregated participants. The GLMs were estimated independently for both the tonal and atonal data and for both frequency bands.

### Brain activity underlying the development of the musical sequences

To determine the temporal evolution of the brain activity underlying musical sequences’ recognition, cluster-based MCS were estimated for five specific time windows that corresponded to each of the five tones comprising the musical sequences. This procedure was carried out independently for both tonal and atonal data and for both frequency bands. Thus, ten cluster-based MCS were calculated for each musical piece (five tones x two frequency bands) on the results of the group-level analysis with an adjusted alpha level of 0.001 (*α* = 0.01/10 = 0.001). This procedure allowed detecting the spatial clusters of significant brain sources underlying the recognition of the tonal and atonal musical sequences. For each of the MCSs, the data were sub-averaged in the time-window of interest (e.g., the time-window for the first tone of the musical sequences) and then submitted to 1000 permutations to build a reference distribution of the maximum cluster sizes detected in the permuted data. Then, using the same procedure as with the MEG channels, the original cluster sizes were compared to the reference distribution and were considered significant if their size was bigger than 99.9% of the maximum cluster sizes of the permuted data.

Importantly, further analyses were conducted to assess the differences between tonal and atonal data when recognizing memorized trials for both the slow and fast frequency bands. For each participant, a *t* test (*α* = 0.01) was computed for each source location and for the five time windows corresponding to each musical tone, contrasting the brain activity underlying the recognition of tonal versus atonal music. Multiple comparisons were corrected for using cluster-based MCS, as described above. In this case, ten MCS (*α* = 0.001, 1000 permutations) were calculated on the significant *t* test results (five tones × two frequency ranges).

### Correlation between familiarity ratings and brain activity underlying the development of the tonal sequences

We examined whether familiarity with J. S. Bach’s Prelude No. 1 in C Major, BWV 846 was correlated with the brain activity during recognition of the tonal sequences extracted from the piece. Outside the scanner, participants were asked to rate their familiarity with the tonal piece on a scale from 1 to 7 (1 = “I never heard it”, 2 = “I occasionally heard it”, 3 = “I sometimes listen to it”, 4 = “I usually listen to it”, 5 = “I played it”, 6 = “I played it in front of an audience”, 7 = “I taught it”).

We computed Pearson’s correlations between participants’ familiarity scores and the brain activity during recognition of each of the five tones comprising the tonal memorized sequences and corrected for multiple comparisons using cluster-based MCS. After obtaining the spatial clusters that significantly correlated (*α* = 0.05) with participants’ familiarity ratings, the data were sub-averaged in five time windows. The significant brain voxels were shuffled in space and the maximum cluster size was measured. Then, we built a reference distribution of the maximum cluster sizes and compared the original cluster sizes to the reference distribution. Clusters were considered significant only if their size was bigger than 95% of the maximum cluster sizes of the permuted data.

### Statistics and reproducibility

The statistical analyses for assessing IC and H levels of the stimuli employed paired-sample *t* tests. The behavioral data were analyzed using two one-way ANOVAs. The MEG sensor data were analyzed using paired-sample *t* tests and corrected for multiple comparisons using cluster-based MCS. The first (subject) level analysis for the MEG source data was calculated using a GLM. The group level was computed using paired-sample *t* tests and corrected for multiple comparisons with cluster-based MCS. The relationship between brain data and previous familiarity with the music was assessed using Pearson’s correlations and corrected for multiple comparisons through cluster-based MCS. Details of these procedures are extensively reported throughout the Methods section. The analyses involved 71 participants.

### Reporting summary

Further information on research design is available in the Nature Research Reporting Summary linked to this article.

## Supplementary information

Supplementary Information

Description of Additional Supplementary Data

Supplementary Data 1

Supplementary Data 2

Supplementary Data 3

Supplementary Data 4

Supplementary Data 5

Supplementary Data 6

Reporting Summary-New

## Data Availability

The neuroimaging data that support the findings of this study have been deposited in Zenodo: https://zenodo.org/record/7249065#.Y1fuES8RppR.

## Code availability

The codes used for the analyses reported in this study are available on GitHub: https://zenodo.org/badge/latestdoi/560818133.
