# Supplementary material for: Magnetoencephalography recordings reveal the spatiotemporal dynamics of recognition memory for complex versus simple auditory sequences
Source: Commun Biol. 2022 Nov 19;5:1272. doi: 10.1038/s42003-022-04217-8 (PMC9675809; doi:10.1038/s42003-022-04217-8)
Supplement: Supplementary file 1 — Supplementary Information [file 42003_2022_4217_MOESM1_ESM.pdf]

Supplementary Information

Supplementary figures

Supplementary Figure 1. Graphical depiction of the significant clusters of activity for tonal MEG sensor data (memorized vs novel musical excerpts)

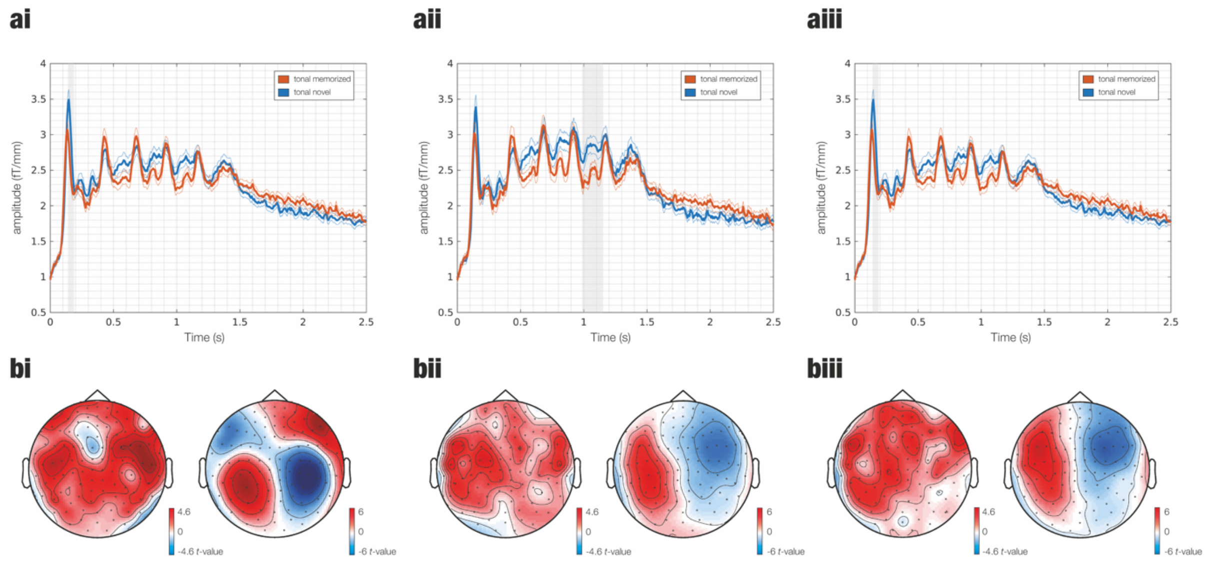

a – The plots represent the signal amplitude of the tonal memorized sequences (in orange) and tonal novel sequences (in blue) in clusters 1 (**i**), 2 (**ii**), and 3 (**iii**). The plots show the full time-window of analyses (0 to 2.5 seconds). Significant time intervals are marked in grey. **b** – The topoplots show the contrast between tonal memorized sequences (in red) and tonal novel sequences (in blue) for each of the significant time intervals of cluster 1 (**i**; 0.14 – 0.187 seconds), 2 (**ii**; 0.987 – 1.153 seconds), and 3 (**iii**; 0.807 – 0.887 seconds). The left topoplots depict the neural activity recorded by gradiometeres and the right topoplots show the neural activity recorded by magnetometers.

Supplementary Figure 2. Graphical depiction of the significant clusters of activity for tonal MEG sensor data (novel vs memorized musical excerpts)

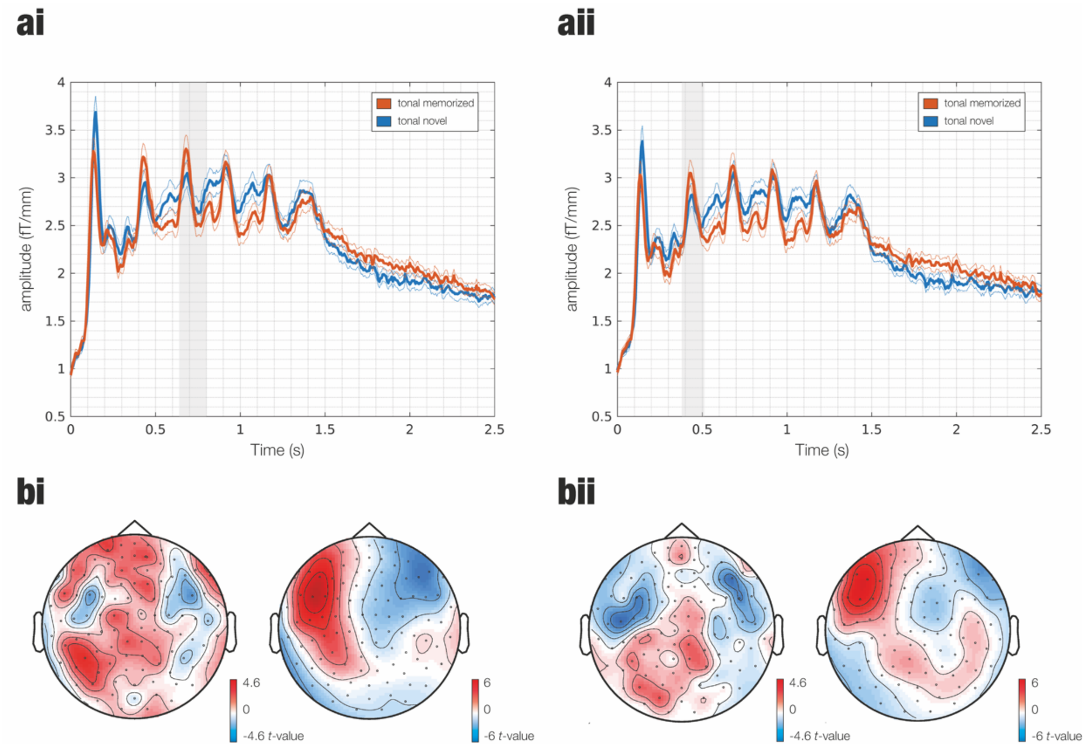

a – The plots represent the signal amplitude of the tonal memorized sequences (in orange) and tonal novel sequences (in blue) in clusters 1 (**i**) and 2 (**ii**). The plots show the full time-window of analyses (0 to 2.5 seconds). Significant time intervals are marked in grey. **b** – The topoplots show the contrast between tonal novel sequences (in red) and tonal memorized sequences (in blue) for each of the significant time intervals of cluster 1 (**i**; 0.64 – 0.8 seconds) and 2 (**ii**; 0.38 – 0.513 seconds). The left topoplots depict the neural activity recorded by gradiometeres and the right topoplots show the neural activity recorded by magnetometers.

Supplementary Figure 3. Graphical depiction of the significant clusters of activity for atonal MEG sensor data (memorized vs novel musical excerpts)

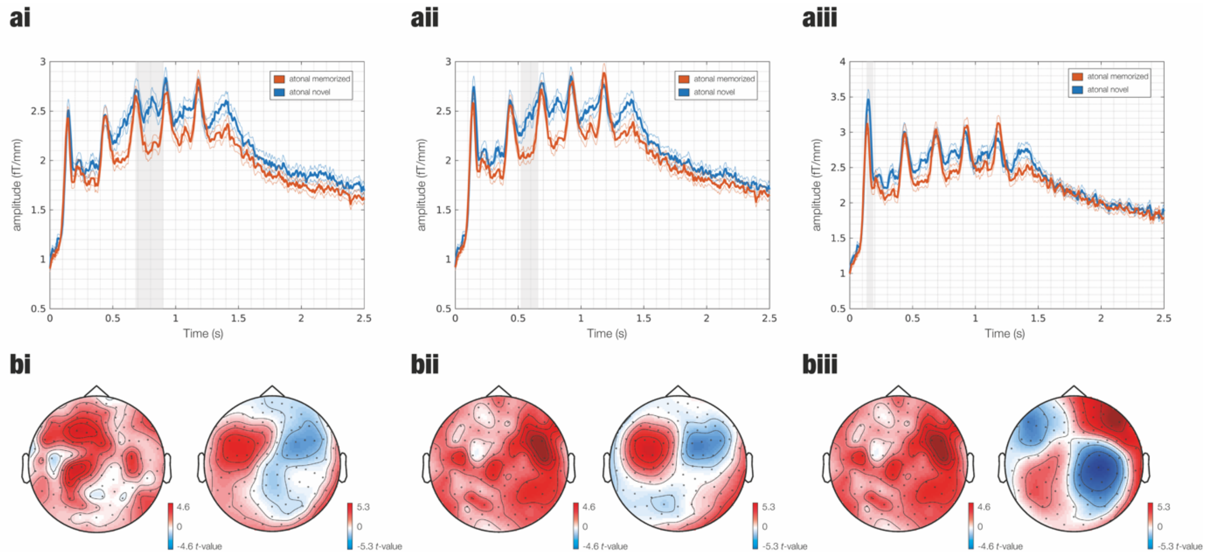

a – The plots represent the signal amplitude of the atonal memorized sequences (in orange) and atonal novel sequences (in blue) in clusters 1 (**i**), 2 (**ii**), and 3 (**iii**). The plots show the full time-window of analyses (0 to 2.5 seconds). Significant time intervals are marked in grey. **b** – The topoplots show the contrast between atonal memorized sequences (in red) and atonal novel sequences (in blue) for each of the significant time intervals of cluster 1 (**i**; 0.68 – 0.9 seconds), 2 (**ii**; 0.52 – 0.66 seconds), and 3 (**iii**; 0.133 – 0.187 seconds). The left topoplots depict the neural activity recorded by gradiometeres and the right topoplots show the neural activity recorded by magnetometers.

Supplementary Figure 4. Graphical depiction of the significant clusters of activity for atonal MEG sensor data (novel vs memorized musical excerpts)

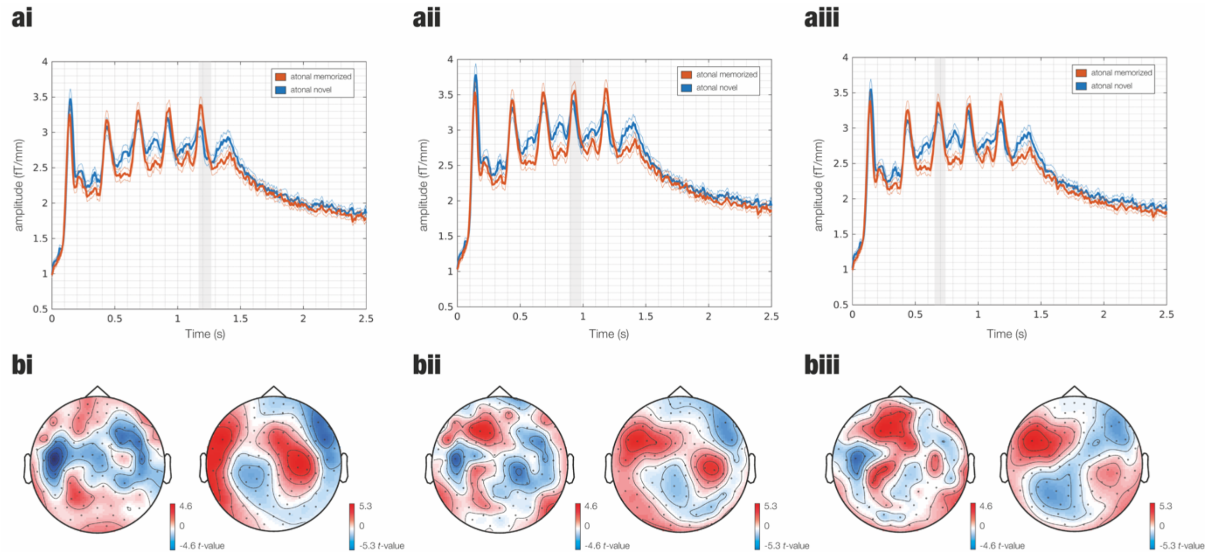

a – The plots represent the signal amplitude of the atonal memorized sequences (in orange) and atonal novel sequences (in blue) in clusters 1 (**i**), 2 (**ii**), and 3 (**iii**). The plots show the full time-window of analyses (0 to 2.5 seconds). Significant time intervals are marked in grey. **b** – The topoplots show the contrast between atonal memorized sequences (in red) and atonal novel sequences (in blue) for each of the significant time intervals of cluster 1 (**i**; 1.167 – 1.267 seconds), 2 (**ii**; 0.893 – 0.987 seconds), and 3 (**iii**; 0.653 – 0.74 seconds). The left topoplots depict the neural activity recorded by gradiometeres and the right topoplots show the neural activity recorded by magnetometers.

Supplementary Figure 5. Brain activity underlying the recognition of musical sequences at 1 – 4 Hz versus 2 – 8 Hz frequency bands

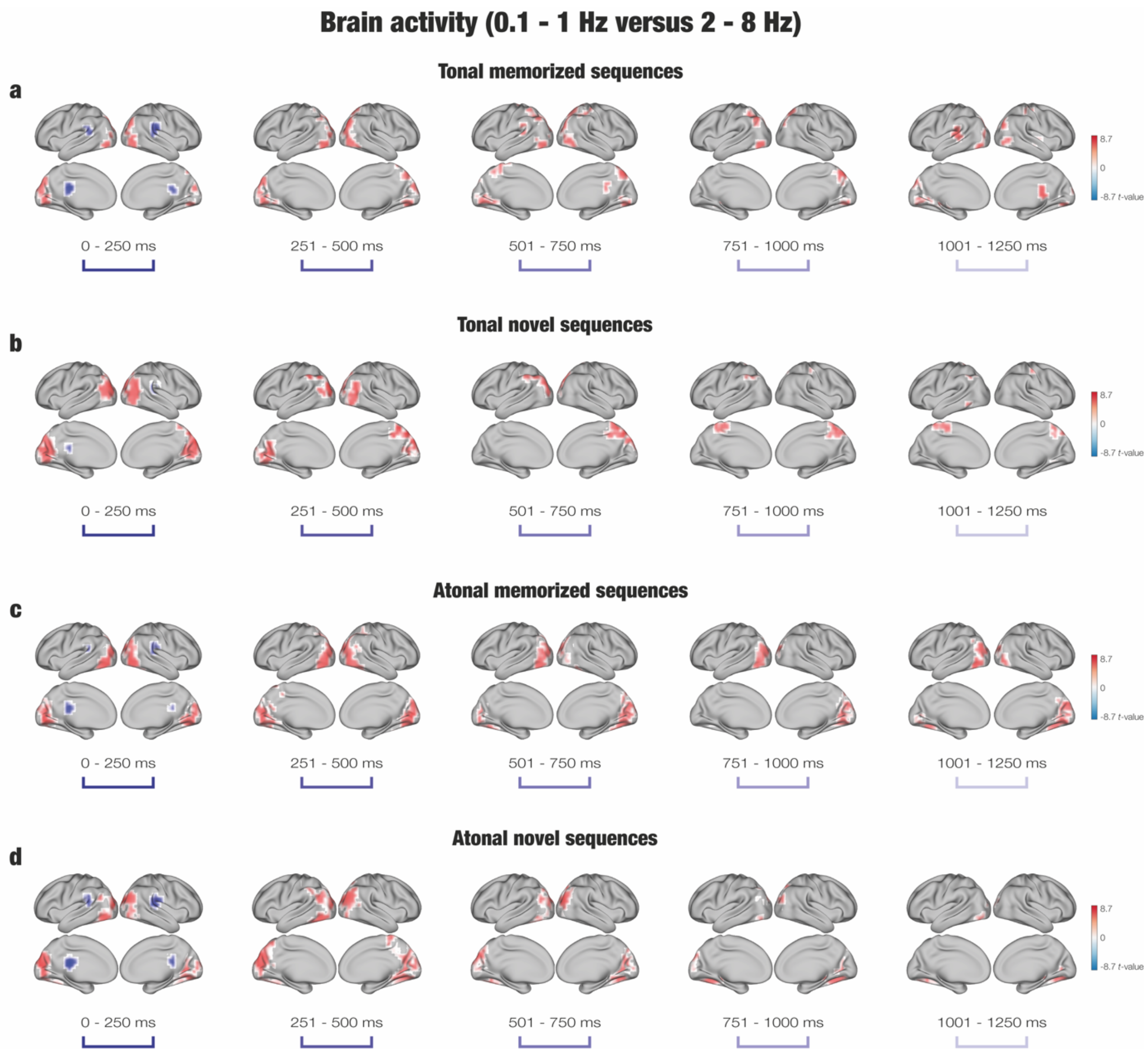

Overall, the brain activity in the slow frequency band (0.1 – 1 Hz, in red) was stronger than in the fast frequency band (2 – 8 Hz, in blue) across all tones and conditions. **a** – For memorized tonal sequences, significant clusters of activity were located in the right superior occipital gyrus and cuneus at the second tone, and in the left superior occipital gyrus, left hippocampus, left angular gyrus, left superior temporal gyrus, and right cuneus at the last three tones of the sequences. The neural activity was stronger for the fast frequency band at the first tone of the sequence, particularly in Heschl's gyrus, insula, Rolandic operculum, and superior temporal gyrus in the right hemisphere. **b** – For tonal novel sequences, the activity for the slow frequency band was localized primarily in the superior parietal gyrus, superior occipital gyrus, cuneus and calcarine fissure in the left hemisphere, and in the precentral gyrus and middle frontal gyrus in the right hemisphere. **c** – For atonal memorized sequences, activity for the fast frequency band was stronger at the first tone in Heschl's gyrus, insula, and temporal gyrus in the right hemisphere. For the slow frequency band, significant clusters were located at the left middle occipital gyrus, right lingual gyrus, and right calcarine fissure at tones two, three, four and five. **d** – For atonal novel sequences, the

activity was again stronger for the fast frequency band at the first tone, particularly in the superior temporal gyrus, insula, Heschl's gyrus, putamen, and Rolandic operculum, in the right hemisphere. For the slow frequency band, significant clusters of activity were localized in the right calcarine fissure and left lingual gyrus.

Supplementary Figure 6. Brain activity underlying the recognition of musical sequences at the 1 – 4 Hz frequency band

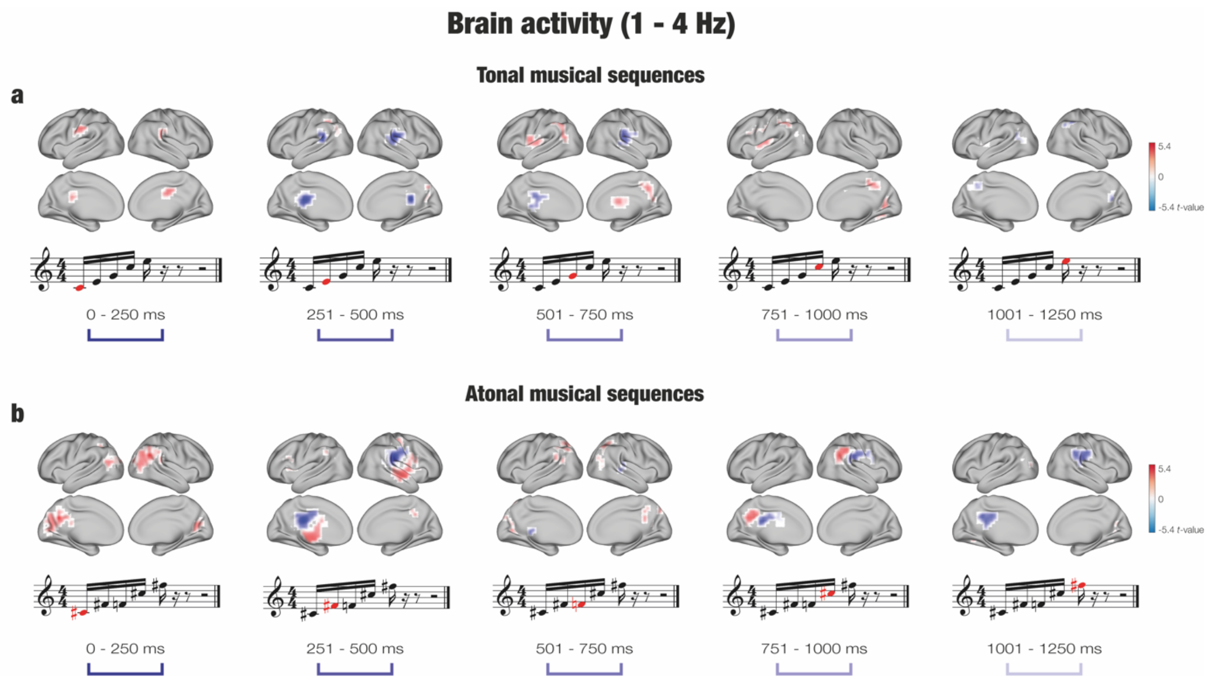

a – For tonal sequences, the brain activity was stronger for memorized (in red) than novel (in blue) sequences, particularly for the first, third, and fourth tones. The difference was localized in the left putamen, left lingual gyrus, left inferior frontal operculum, and right Heschl's gyrus. The brain activity was stronger for novel than memorized sequences for the second tone in the left superior temporal gyrus, right insula, and right putamen. **b** – For atonal sequences, the brain activity was stronger for memorized than novel sequences for the last first, second, and third tones in areas such as the right precuneus and right calcarine fissure. The brain activity was stronger for novel than memorized sequences for the last two tones in the right Heschl's gyrus and right Rolandic operculum.

Supplementary Figure 7. Brain activity underlying the recognition of musical sequences at the 5 – 8 Hz frequency band

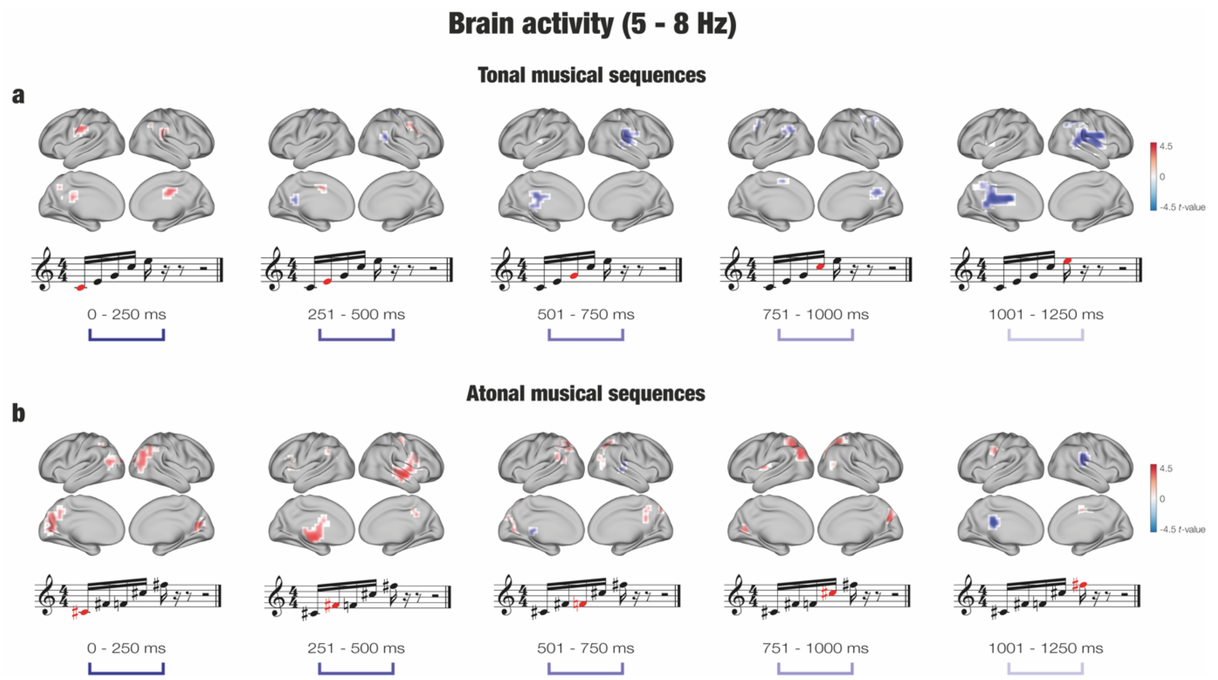

a – For tonal sequences, the brain activity was stronger for novel (in blue) than memorized (in red) sequences, particularly for the second, third, fourth, and fifth tones. The difference was localized in the right inferior frontal gyrus, left caudate, and right insula. The brain activity was stronger for memorized than novel sequences for the first tone at the left inferior frontal gyrus and right Heschl's gyrus. **b** – For atonal sequences, the brain activity was stronger for memorized than novel sequences for the first four tones, particularly in the left calcarine fissure and right precuneus. The brain activity was stronger for novel than memorized sequences for the last tone in the right insula and right putamen.

Supplementary Figure 8. Brain activity underlying the correlation between familiarity ratings and recognition of tonal musical sequences

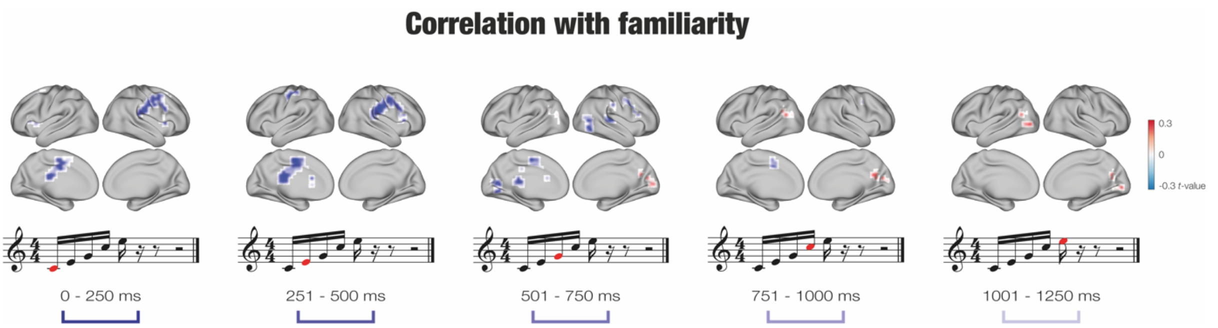

Significant Pearson's correlations between the brain activity underlying recognition of the tonal sequences and familiarity ratings. The correlations are depicted in brain templates in five subsequent time windows corresponding to the duration of each musical tone forming the sequences. The color bar shows Pearson's correlation coefficient obtained by correlating the brain activity underlying recognition of the memorized tonal sequences with the familiarity ratings.
