# Supplementary material for: Magnetoencephalography recordings reveal the spatiotemporal dynamics of recognition memory for complex versus simple auditory sequences
Source: Commun Biol. 2022 Nov 19;5:1272. doi: 10.1038/s42003-022-04217-8 (PMC9675809; doi:10.1038/s42003-022-04217-8)
Supplement: Supplementary file 2 — Description of Additional Supplementary Data [file 42003_2022_4217_MOESM2_ESM.docx]

**Description of Additional Supplementary Files**

**File name:** Supplementary Data 1

**Title:** Numerical source data for behavioral responses

**Description:** Response accuracy and reaction time (in milliseconds) for each of the four experimental conditions (tonal memorized, tonal novel, atonal memorized, and atonal novel).

**File name:** Supplementary Data 2

**Title:** Significant clusters of activity for MEG sensor data

**Description:** Significant clusters of activity estimated from the two-sided contrasts between memorized and novel musical sequences. This was performed for both tonal and atonal sequences independently. The table depicts the number of significant clusters, along with the MEG channels and time-windows.

**File name:** Supplementary Data 3

**Title:** Significant clusters of activity for MEG source data

**Description:** Significant clusters of activity estimated from the contrasts between tonal and atonal memorized musical sequences. This was performed for both delta and theta frequency bands independently. The table depicts the contrast for each of the tones comprising the musical sequences, along with the brain regions, hemispheres, and averaged t-values for each voxel

**File name:** Supplementary Data 4

**Title:** Significant clusters of activity for MEG source data (0.1 – 1 Hz versus 2 – 8 Hz frequency bands).

**Description:** Significant clusters of activity estimated from the contrasts between the slow (0.1 – 1 Hz) and fast (2 – 8Hz) frequency bands for tonal memorized, tonal novel, atonal memorized, and atonal novel sequences independently. The table depicts the contrast for each of the tones comprising the musical sequences, along with the brain regions, hemispheres, and averaged t-values for each voxel.

**File name:** Supplementary Data 5

**Title:** Significant clusters of activity for MEG source data (1 – 4 Hz and 5 – 8 Hz frequency bands)

**Description:** Significant clusters of activity estimated from the contrasts between memorized and novel musical sequences for both tonal and atonal data independently. This was performed for the 1 – 4 Hz and 5 – 8 Hz frequency bands. The table depicts the contrast for each of the tones comprising the musical sequences, along with the brain regions, hemispheres, and averaged t-values for each voxel.

**File name:** Supplementary Data 6

**Title:** Significant clusters emerged from the correlation between familiarity ratings and MEG brain data underlying recognition of previously memorized tonal musical sequences.

**Description:** Significant clusters of activity estimated from the correlations between familiarity ratings and the brain activity (in 0.1 – 1 Hz) underlying previously memorized tonal musical sequences. The table depicts the correlation for each of the tones comprising the musical sequences, along with the brain regions, hemispheres, and r-values for each voxel.
