# Supplementary material for: Magnetoencephalography recordings reveal the spatiotemporal dynamics of recognition memory for complex versus simple auditory sequences
Source: Commun Biol. 2022 Nov 19;5:1272. doi: 10.1038/s42003-022-04217-8 (PMC9675809; doi:10.1038/s42003-022-04217-8)
Supplement: Supplementary file 9 — Reporting Summary-New [file 42003_2022_4217_MOESM9_ESM.pdf]

nature portfolio

Corresponding author(s): Gemma Fernández Rubio

Last updated by author(s): Oct 25, 2022

Reporting Summary

Nature Portfolio wishes to improve the reproducibility of the work that we publish. This form provides structure for consistency and transparency in reporting. For further information on Nature Portfolio policies, see our Editorial Policies and the Editorial Policy Checklist.

Statistics

For all statistical analyses, confirm that the following items are present in the figure legend, table legend, main text, or Methods section.

n/a	Confirmed	
☐	☒	The exact sample size (*n*) for each experimental group/condition, given as a discrete number and unit of measurement
☐	☒	A statement on whether measurements were taken from distinct samples or whether the same sample was measured repeatedly
☐	☒	The statistical test(s) used AND whether they are one- or two-sided<br>*Only common tests should be described solely by name; describe more complex techniques in the Methods section.*
☒	☐	A description of all covariates tested
☐	☒	A description of any assumptions or corrections, such as tests of normality and adjustment for multiple comparisons
☐	☒	A full description of the statistical parameters including central tendency (e.g. means) or other basic estimates (e.g. regression coefficient) AND variation (e.g. standard deviation) or associated estimates of uncertainty (e.g. confidence intervals)
☐	☒	For null hypothesis testing, the test statistic (e.g. *F*, *t*, *r*) with confidence intervals, effect sizes, degrees of freedom and *P* value noted<br>*Give P values as exact values whenever suitable.*
☒	☐	For Bayesian analysis, information on the choice of priors and Markov chain Monte Carlo settings
☐	☒	For hierarchical and complex designs, identification of the appropriate level for tests and full reporting of outcomes
☐	☒	Estimates of effect sizes (e.g. Cohen's *d*, Pearson's *r*), indicating how they were calculated

Our web collection on statistics for biologists contains articles on many of the points above.

Software and code

Policy information about availability of computer code

Data collection	Elekta Neuromag TRIUX system (Elekta Neuromag, Helsinki, Finland)<br>Polhemus Fastrak, Colchester, VT, USA<br>Presentation software (Neurobehavioural Systems, Berkeley, CA)
Data analysis	MaxFilter<br>Matlab (MathWorks, Natick, Massachusetts, United States of America)

For manuscripts utilizing custom algorithms or software that are central to the research but not yet described in published literature, software must be made available to editors and reviewers. We strongly encourage code deposition in a community repository (e.g. GitHub). See the Nature Portfolio guidelines for submitting code & software for further information.

Data

Policy information about availability of data

All manuscripts must include a data availability statement. This statement should provide the following information, where applicable:

- Accession codes, unique identifiers, or web links for publicly available datasets
- A description of any restrictions on data availability
- For clinical datasets or third party data, please ensure that the statement adheres to our policy

The neuroimaging data that support the findings of this study have been deposited in Zenodo: https://zenodo.org/record/7249065#.Y1fuES8RppR.

Human research participants

Policy information about studies involving human research participants and Sex and Gender in Research.

Reporting on sex and gender	Sex was determined based on self-reporting. Sex-based analyses were not performed because this variable was not considered relevant to the experimental hypotheses, but the sample recruited was balanced in terms of sex (53.52% male, 46.47% female).
Population characteristics	The population consisted of 71 volunteers (38 males and 33 females) aged 18 to 42 years old (mean age: 25 ± 4.10 years). All participants were healthy and reported normal hearing. Participants were recruited in Denmark and came from Western countries with matching socioeconomic and educational backgrounds.
Recruitment	Participants were recruited via social media, online databases, and printed flyers.
Ethics oversight	Ethics Committee of the Central Denmark Region (De Videnskabsetiske Komitéer for Region Midtjylland) (Ref 1-10-72-411-17)

Note that full information on the approval of the study protocol must also be provided in the manuscript.

Field-specific reporting

Please select the one below that is the best fit for your research. If you are not sure, read the appropriate sections before making your selection.

☒ Life sciences ☐ Behavioural & social sciences ☐ Ecological, evolutionary & environmental sciences

For a reference copy of the document with all sections, see nature.com/documents/nr-reporting-summary-flat.pdf

Life sciences study design

All studies must disclose on these points even when the disclosure is negative.

Sample size	The sample size was selected based on previous analogous studies.
Data exclusions	No data were excluded from the analyses.
Replication	This is not a replication study, but the results reported are coherent with previous literature using a similar methodology (e.g., standard preprocessing pipeline and beamforming algorithms for the magnetoencephalography source reconstruction).
Randomization	Participants were randomly selected from a pool of people interested in participating in the study.
Blinding	Blinding was not applicable in this study, since there was only one group of participants.

Reporting for specific materials, systems and methods

We require information from authors about some types of materials, experimental systems and methods used in many studies. Here, indicate whether each material, system or method listed is relevant to your study. If you are not sure if a list item applies to your research, read the appropriate section before selecting a response.

Materials & experimental systems

n/a	Involved in the study	
☒	☐	Antibodies
☒	☐	Eukaryotic cell lines
☒	☐	Palaeontology and archaeology
☒	☐	Animals and other organisms
☒	☐	Clinical data
☒	☐	Dual use research of concern

Methods

n/a	Involved in the study	
☒	☐	ChIP-seq
☒	☐	Flow cytometry
☐	☒	MRI-based neuroimaging

Magnetic resonance imaging

Experimental design

Design type: Resting state

Design specifications: One block lasting 20 minutes

Behavioral performance measures: N/A

Acquisition

Imaging type(s): Structural

Field strength: T1

Sequence & imaging parameters: The data were recorded with a spatial resolution of 1.0 x 1.0 x 1.0 mm and the following sequence parameters were applied: echo time (TE) = 2.96 ms, repetition time (TR) = 5000 ms, reconstructed matrix size = 256 x 256, bandwidth = 240 Hz/Px.

Area of acquisition: Whole brain scan

Diffusion MRI: ☐ Used ☒ Not used

Preprocessing

Preprocessing software: Statistical Parametric Mapping (SPM) and FMRIB Software Library (FSL)

Normalization: Each individual T1-weighted MRI scan was successively co-registered to the standard Montreal Neurological Institute (MNI) brain template through an affine transformation and then referenced to the magnetoencephalography (MEG) sensors space by using the Polhemus head shape data and the three fiducial points measured during the MEG session.

Normalization template: Montreal Neurological Institute (MNI) brain template (MNI152 T1)

Noise and artifact removal: The structural T1 images used for magnetoencephalography (MEG) source localization purposes were clean enough after normalization and coregistration with MNI templates and MEG fiducials data. Thus, no additional noise-reduction algorithms were used.

Volume censoring: N/A

Statistical modeling & inference

Model type and settings: N/A

Effect(s) tested: N/A

Specify type of analysis: ☐ Whole brain ☐ ROI-based ☐ Both

Statistic type for inference (See Eklund et al. 2016): N/A

Correction: N/A

Models & analysis

n/a	Involved in the study
☒	☐ Functional and/or effective connectivity
☒	☐ Graph analysis
☒	☐ Multivariate modeling or predictive analysis
